# IgGFc-binding protein in pregnancies complicated by spontaneous preterm delivery: a retrospective cohort study

**DOI:** 10.1038/s41598-021-85473-2

**Published:** 2021-03-17

**Authors:** Jaroslav Stranik, Marian Kacerovsky, Ondrej Soucek, Martina Kolackova, Ivana Musilova, Lenka Pliskova, Radka Bolehovska, Pavel Bostik, Jana Matulova, Bo Jacobsson, Ctirad Andrys

**Affiliations:** 1 The Department of Obstetrics and Gynecology, University Hospital Hradec Kralove, Charles University, Faculty of Medicine in Hradec Kralove, Sokolska 581, 500 05 Hradec Králové, Czech Republic; 2 Biomedical Research Center, University Hospital Hradec Kralove, Hradec Králové, Czech Republic; 3 Department of Clinical Immunology and Allergy, University Hospital Hradec Kralove, Charles University, Faculty of Medicine in Hradec Kralove, Hradec Králové, Czech Republic; 4 Institute of Clinical Biochemistry and Diagnostics, University Hospital Hradec Kralove, Charles University, Faculty of Medicine in Hradec Kralove, Hradec Králové, Czech Republic; 5 Institute of Clinical Microbiology, University Hospital in Hradec Kralove, Charles University, Faculty of Medicine Hradec Kralove, Hradec Králové, Czech Republic; 6 Department of Social Medicine, Charles University, Faculty of Medicine in Hradec Kralove, Hradec Králové, Czech Republic; 7 Department of Obstetrics and Gynecology, Institute of Clinical Science, Sahlgrenska Academy, University of Gothenburg, Gothenburg, Sweden; 8 Department of Obstetrics and Gynecology, Region Västra Götaland, Sahlgrenska University Hospital, Gothenburg, Sweden; 9 Department of Genetics and Bioinformatics, Domain of Health Data and Digitalisation, Institute of Public Health, Oslo, Norway

**Keywords:** Diagnostic markers, Urogenital reproductive disorders, Infection

## Abstract

To determine the IgGFc-binding protein (FcgammaBP) concentration in amniotic and cervical fluids in preterm prelabor rupture of membranes (PPROM) and preterm labor with intact membranes (PTL) and to assess the diagnostic indices of FcgammaBP to predict intra-amniotic infection (the presence of both microbial invasion of the amniotic cavity and intra-amniotic inflammation). In this study, we included 170 and 79 women with PPROM and PTL, respectively. Paired cervical and amniotic fluid samples were obtained using a Dacron polyester swab and transabdominal amniocentesis, respectively. The FcgammaBP concentrations in the samples were assessed using an enzyme-linked immunosorbent assay. The presence of intra-amniotic infection was associated with elevated FcgammaBP concentrations in pregnancies with PPROM and PTL [PPROM—presence: 86 ng/mL vs. absence: 13 ng/mL, *p* < 0.0001, area under receiver operating characteristic curve (AUC) = 0.94; PTL—presence: 140 ng/mL vs. absence: 22 ng/mL, *p* < 0.0001, AUC = 0.86]. In cervical fluid, the concentrations of FcgammaBP were elevated in the presence of intra-amniotic infection in pregnancies with PPROM only (presence: 345 ng/mL vs. absence: 60 ng/mL, *p* < 0.0001, AUC = 0.93). FcgammaBP in amniotic fluid might be a marker of intra-amniotic infection in women with both PPROM and PTL However, in cervical fluid, it is only observed in women with PPROM.

## Introduction

Preterm delivery, defined as delivery before 37 weeks of gestation, is divided into two major subgroups: (i) iatrogenic preterm delivery and (ii) spontaneous preterm delivery^1,2^. The latter form of preterm delivery is more frequent and is responsible for more than two-thirds of all preterm deliveries^1,2^. Spontaneous preterm delivery can also be divided into two clinical phenotypes: (i) preterm prelabor rupture of the membranes (PPROM) and (ii) preterm labor with intact membranes (PTL)^1,2^.

Some pregnancies can be complicated by the elevation of amniotic fluid concentrations of various inflammatory mediators, such as cytokines, chemokines, and antimicrobial peptides^3–11^. This condition is termed intra-amniotic inflammation and can be identified in both PPROM^12,13^ and PTL^14^. Based on the presence or absence of microorganisms and/or their nucleic acids in amniotic fluid, intra-amniotic inflammation can be further divided into two clinical subtypes: (i) intra-amniotic infection and (ii) sterile intra-amniotic inflammation^12,14,15^. Clinical relevance of intra-amniotic inflammation, its association with adverse short- and long-term neonatal outcomes, and optimal diagnostic markers, are still a matter of intense debate^16–28^.

The broad availability and recent advances in proteomics, an unbiased technology, bring it within the scope of researchers working in the field of intra-amniotic inflammatory complications^29–38^. The ability to identify hundreds of proteins and to quantify changes in their abundance across multiple amniotic fluid samples makes proteomics very appealing, particularly in the discovery phase of the biomarker search process^29–61^. Therefore, proteomic analysis of amniotic fluid may reveal new proteins involved in the complex pathogenesis of intra-amniotic inflammation^29–40^.

IgGFc-binding protein (FcgammaBP) is one of the proteins identified in amniotic fluid using proteomics^36,37,40,42^. FcgammaBP is a relatively unknown protein, with limited reports in relation to conditions such as bowel inflammatory disease, autoimmune disease, or thyroid gland tumors^62–64^. Nevertheless, FcgammaBP has been identified in amniotic fluid obtained from women with uncomplicated pregnancies^40,42^ as well as from women with pregnancies complicated by PPROM^36^ and PTL^37^. In pregnancies with PPROM, an elevation of FcgammaBP concentration in amniotic fluid has been observed during microbial invasion of the amniotic cavity and in acute histological chorioamnionitis^36^. However, there is a paucity of information on whether concentrations of FcgammaBP in amniotic and cervical fluid reflect the presence of intra-amniotic inflammatory complications in both the clinical phenotypes of spontaneous preterm delivery.

To fill this gap in the knowledge, we conducted this study with the following goals: (i) to quantify the FcgammaBP concentration in amniotic fluid samples from pregnant women with PPROM and PTL based on the phenotype of intra-amniotic inflammation; (ii) to quantify the FcgammaBP concentration in cervical fluid samples from pregnant women with PPROM and PTL based on the phenotype of intra-amniotic inflammation; and (iii) to assess the predictive value of FcgammaBP concentrations in amniotic and cervical fluids for intra-amniotic infection in pregnant women with PPROM and PTL.

## Results

In total, 170 and 79 women with PPROM and PTL, respectively were included in the study. Among women with PPROM, the presence of intra-amniotic infection, sterile intra-amniotic inflammation, colonization, and negative amniotic fluid were observed in 11% (19/170), 5% (9/170), 10% (16/170), and 74% (126/170) of women, respectively. Among women with PTL, intra-amniotic infection, sterile intra-amniotic inflammation, and negative amniotic fluid were found in 15% (12/79), 27% (21/79), and 58% (46/79) of the women, respectively. None of the women with PTL had colonization.

The demographics of all the women in this study and clinical data of the women with PPROM and PTL, based on the presence and absence of intra-amniotic infection, are shown in Tables 1 and 2, respectively. The microorganisms identified in the amniotic fluid from women with PPROM and PTL are listed in Table 3.

**Table 1:** Maternal and clinical characteristics of women with preterm prelabor rupture of membranes based on the presence and absence of intra-amniotic infection.

Characteristic	The presence of intra-amniotic infection (n = 19)	The absence of intra-amniotic infection (n = 151)	*p-*value
Maternal age [years, median (IQR)]	30 (24–33)	31 (27–35)	0.19
Primiparous [number (%)]	10 (53%)	90 (60%)	0.63
Pre-pregnancy body mass index [kg/m^2^, median (IQR)]	22.8 (20.9–25.2)	24.0 (21.2–27.2)	0.25
Smoking [number (%)]	3 (16%)	25 (17%)	1.00
Interval between PPROM and amniocentesis [hours, median (IQR)]	3 (2–8)	4 (2–7)	0.57
Gestational age at admission [weeks, median (IQR)]	29 + 3 (27 + 0–33 + 4)	34 + 3 (32 + 3–35 + 3)	**0.0003**
Gestational age at delivery [weeks, median (IQR)]	29 + 6 (27 + 3–33 + 4)	34 + 5 (33 + 0–35 + 6)	** < 0.0001**
Latency between PPROM and delivery [hours, median (IQR)]	60 (17–113)	45 (13–157)	0.70
Amniotic fluid IL-6 levels at admission [pg/nL, median (IQR)]	30,932 (9427–50,000)	667 (329–50,000)	** < 0.0001**
CRP levels at admission [mg/L, median (IQR)]	19.2 (3.3–36.5)	5.2 (2.5–8.2)	**0.003**
WBC count at admission [×10^9^ L, median (IQR)]	14.9 (11.2–17.6)	11.9 (9.9–14.3)	**0.03**
Administration of corticosteroids [number (%)]	15 (79%)	89 (59%)	0.13
Vaginal delivery [number (%)]	13 (68%)	108 (72%)	0.79
Birth weight [grams, median (IQR)]	1460 (1090–2220)	**2290 (1930–3620)**	** < 0.0001**
Apgar score < 7; 5 min [number (%)]	3 (16%)	5 (3%)	**0.05**
Apgar score < 7; 10 min [number (%)]	2 (11%)	2 (1%)	0.06

Continuous variables were compared using a nonparametric Mann–Whitney *U* test. Categorical variables were compared using the Fisher’s exact test.

Continuous variables are presented as median (IQR) and categorical as number (%).

Statistically significant results are marked in bold.

CRP: C-reactive protein; IL-6: interleukin-6; IQR: interquartile range; WBC: white blood cells.

**Table 2:** Maternal and clinical characteristics of women with spontaneous preterm labor with intact membranes based on the presence and absence of intra-amniotic infection.

Characteristic	The presence of intra-amniotic infection (n = 12)	The absence of intra-amniotic infection (n = 67)	*p*-value
Maternal age [years, median (IQR)]	27 (25–28)	28 (23–30)	0.86
Primiparous [number (%)]	7 (58%)	47 (70%)	0.50
Pre-pregnancy body mass index [kg/m^2^, median (IQR)]	27.5 (23.1–30.6)	25.0 (23.0–27.9)	0.51
Smoking [number (%)]	1 (8%)	7 (10%)	1.00
Gestational age at admission [weeks, median (IQR)]	27 + 6 (26 + 6–31 + 2)	30 + 6 (26 + 6–32 + 3)	0.17
Gestational age at delivery [weeks, median (IQR)]	29 + 0 (27 + 1–33 + 3)	32 + 6 (29 + 0–36 + 5)	**0.04**
Interval from amniocentesis to delivery [days, median (IQR)]	2 (0–15)	5 (1–39)	0.10
Amniotic fluid IL-6 levels at admission [pg/mL, median (IQR)]	43,431 (23,597–50,000)	1495 (484–4050)	** < 0.0001**
CRP levels at admission [mg/L, median (IQR)]	42.0 (7.5–75.1)	6.0 (2.4–11.4)	**0.0007**
WBC count at admission [×10^9^ L, median (IQR)]	16.3 (14.1–19.5)	13.9 (10.6–16.5)	0.05
Administration of corticosteroids [number (%)]	9 (75%)	57 (85%)	0.41
Vaginal delivery [number (%)]	9 (75%)	55 (82%)	0.69
Birth weight of the newborn [grams, median (IQR)]	1230 (936–1958)	1940 (130–2690)	**0.03**
Apgar score < 7; 5 min [number (%)]	3 (25%)	7 (10%)	0.17
Apgar score < 7; 10 min [number (%)]	2 (17%)	3 (5%)	0.16

Continuous variables were compared using a nonparametric Mann–Whitney *U* test. Categorical variables were compared using the Fisher’s exact test. Continuous variables are presented as median (IQR) and categorical as number (%).

Statistically significant results are marked in bold.

RP: C-reactive protein; IL: interleukin; IQR: interquartile range; WBC: white blood cells.

**Table 3:** The microbial species identified in the amniotic fluid of women with preterm prelabor rupture of membranes and with spontaneous preterm labor with intact membranes.

Preterm prelabor rupture of membranes	Preterm labor with intact membranes
*Ureaplasma* spp. (n = 18)	*Ureaplasma* spp. (n = 4)
*Ureaplasma* spp. + *Mycoplasma hominis* (n = 1)	*Klebsiella pneumoniae* + *Streptococcus anginosus* (n = 1)
*Ureaplasma* spp. + *Gardnerella vaginalis* (n = 1)	*Gardnerella vaginalis* + *Lactobacillus plantum* (n = 1)
*Ureaplasma* spp. + *Streptococcus mitis* (n = 1)	*Ureaplasma* spp. + *Mycoplasma hominis* (n = 1)
*Ureaplasma* spp. + *Escherichia coli* (n = 1)	*Haemophilus influenzae* (n = 1)
*Ureaplasma* spp. + *Fusobacterium nucleatum* v	*Lachnoanaerobaculum* spp. (n = 1)
*Ureaplasma* spp. + *Dialister micraerophilus* + *Atopobium vaginae* (n = 1)	*Lactococcus lactis* (n = 1)
*Gardnerella vaginalis* + *Sneathia sanguinegens* (n = 1)	*Sneathia sanguinegens* (n = 1)
*Haemophilus influenzae* (n = 4)	Non-identifiable bacteria by sequencing (n = 1)
*Anaerococcus tetradius* (n = 1)	
*Chlamydia trachomatis* (n = 1)	
*Lactobacillus iners* (n = 1)	
*Lactobacillus jensenii* (n = 1)	
*Mycoplasma hominis* (n = 1)	
*Streptococcus anginosus* (n = 1)	

### Amniotic fluid FcgammaBP concentrations based on the phenotype of intra-amniotic inflammation

#### PPROM pregnancies

**Figure 1:**
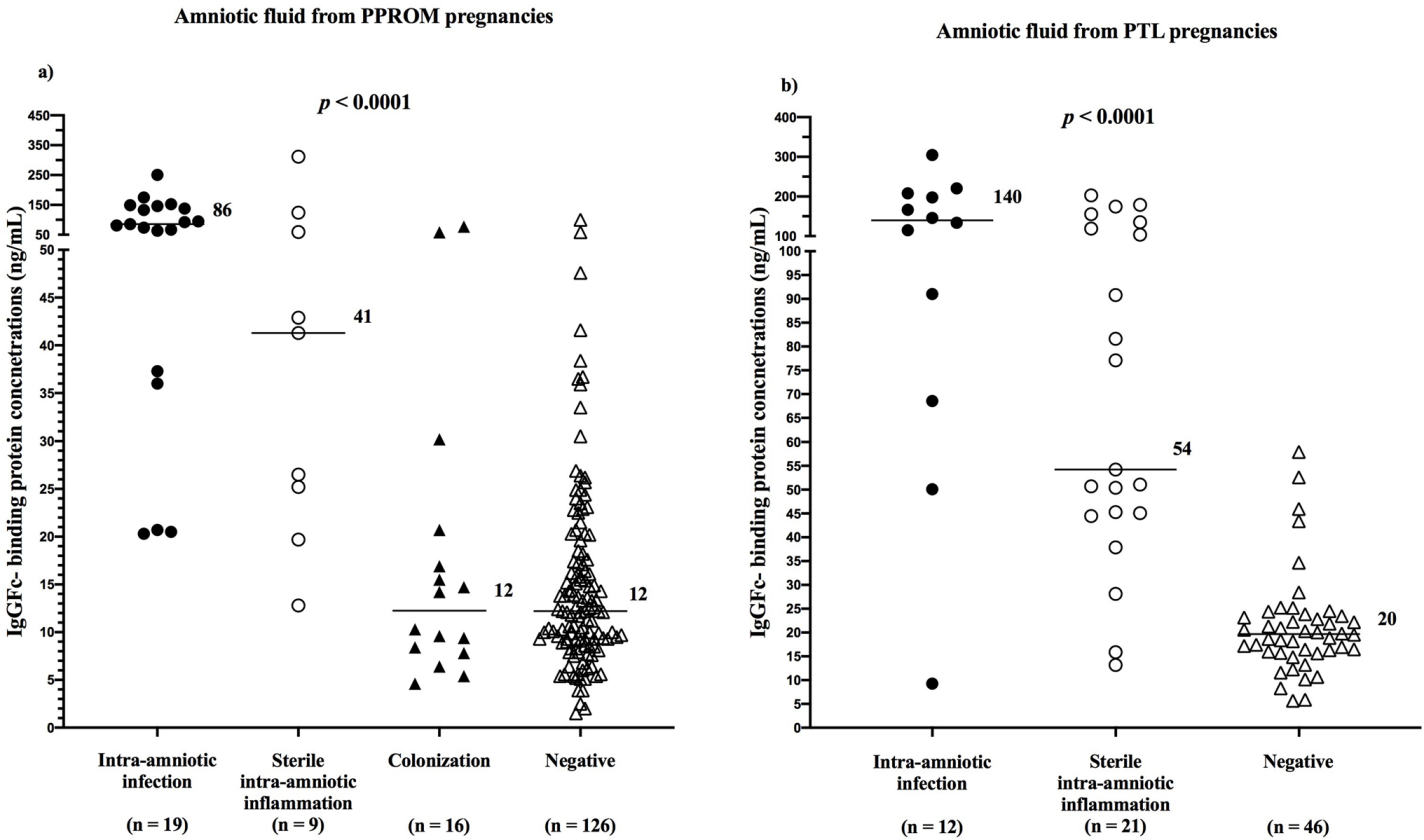
Amniotic fluid IgGFc-binding protein concentrations in the subgroups of the women with PPROM (**a**) and with PTL (**b**). PPROM, preterm prelabor rupture of membranes; PTL, preterm labor with intact membranes.

PPROM pregnancies with intra-amniotic infection and sterile intra-amniotic inflammation had higher amniotic fluid FcgammaBP concentrations than did the women with colonization and with negative amniotic fluid (infection: median 85.6 ng/mL, IQR 37.3–146.0, sterile: median 41.3 ng/mL, IQR 22.5–91.4, colonization: median 12.3 ng/mL, IQR 8.0–19.8, negative 12.2 ng/mL, IQR 9.0–17.8; *p* < 0.0001; Fig. 1a). No differences in amniotic fluid FcgammaBP concentrations were found between the women with colonization and negative amniotic fluid (Table 4).

**Table 4:** IgGFc-binding protein in amniotic fluid from preterm prelabor rupture of membranes: the comparisons among the subgroups of the women with intra-amniotic infection, sterile intra-amniotic inflammation, colonization, and negative amniotic fluid.

	Intra-amniotic infection	Sterile intra-amniotic inflammation	Colonization	Negative
Intra-amniotic infection	x	*p* = 0.11 adj. *p* = 0.51	*p* < 0.0001 **adj. p < 0.0001**	*p* < 0.0001 **adj. p < 0.0001**
Sterile intra-amniotic inflammation	*p* = 0.11 adj. *p* = 0.51	x	*p* = 0.004 **adj. p = 0.05**	*p* < 0.0001 **adj. p < 0.0001**
Colonization	*p* < 0.0001 **adj. p = 0.0001**	*p* = 0.004 **adj. p = 0.05**	x	*p* = 0.98 adj. *p* = 0.22
Negative	*p* < 0.0001 **adj. p < 0.0001**	*p* < 0.0001 **adj. p < 0.0001**	*p* = 0.98 adj. *p* = 0.22	x

*p*-value: a comparison between two subgroups (a nonparametric Mann–Whitney *U* test);

Adj. *p*-value: a comparison between two subgroups after the adjustment for gestational age at sampling (a Spearman partial correlation).

Statistically significant results are marked in bold.

**Figure 2:**
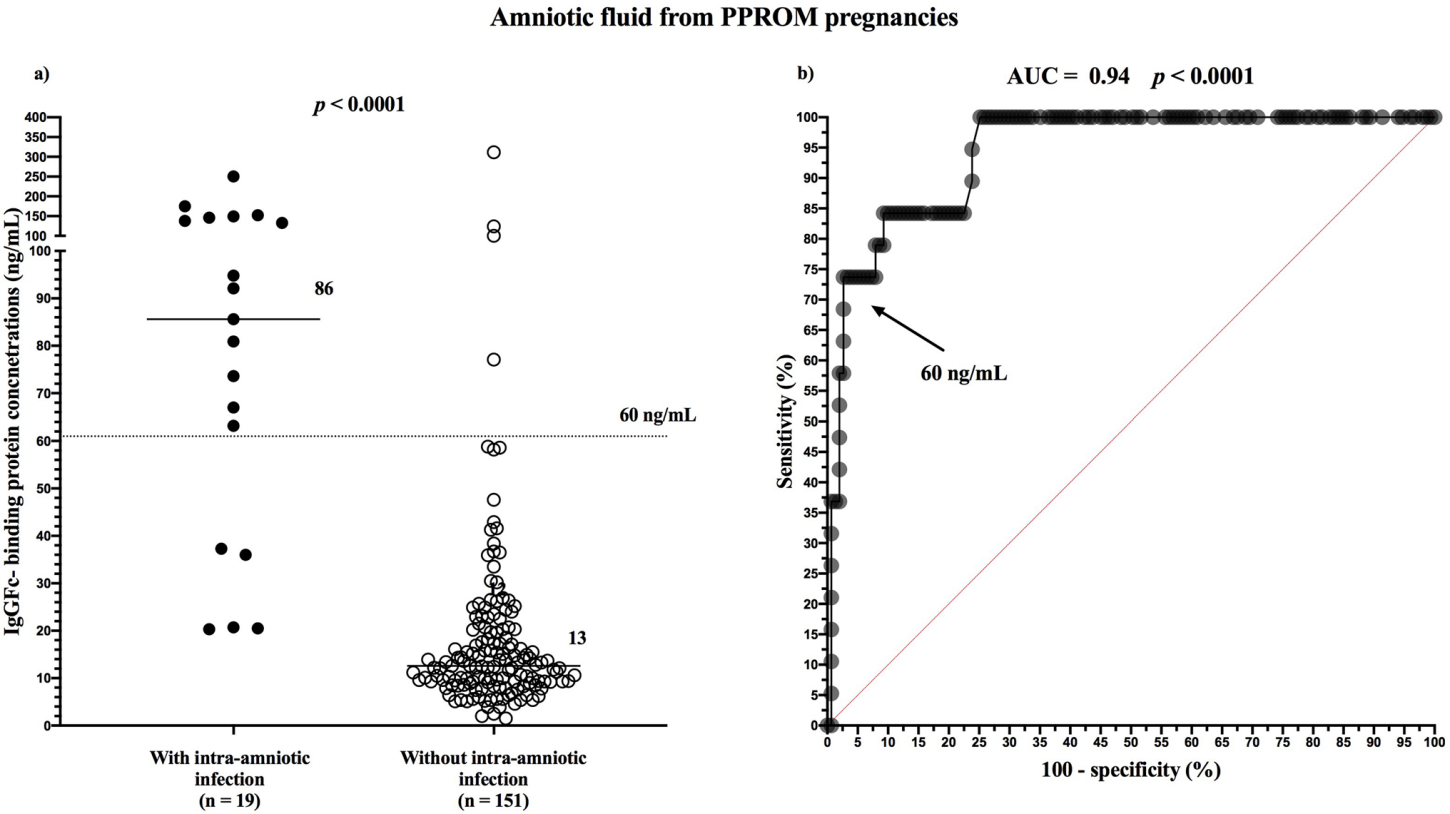
Amniotic fluid IgGFc-binding protein concentrations based on the presence of intra-amniotic infection in women with PPROM (**a**) and receiver operating characteristic curves for amniotic fluid IgGFc-binding protein in women with PPROM with intra-amniotic infection (**b**). PPROM, preterm prelabor rupture of membranes.

Women with intra-amniotic infection had higher amniotic fluid FcgammaBP than did those without intra-amniotic infection (with infection: median IQR 85.6 ng/mL, IQR 37.3–146.0 vs. without infection: median 12.6 ng/mL, IQR 9.1–20.3; Fig. 2a). The amniotic fluid FcgammaBP cutoff value of 60 ng/mL was optimal in the prediction of intra-amniotic infection (area under the ROC curve [AUC] = 0.94; *p* < 0.0001; Fig. 2b). The diagnostic indices of these cutoff values are in Table 5.

**Table 5:** The predictive values of cut-off values of amniotic fluid IgGFcBP to identify intra-amniotic infection.

Cut-off value	Sensitivity	Specificity	Positive predictive value	Negative predictive value	Positive likelihood ratio	Negative likelihood ratio	Odds ratio
**Preterm prelabor rupture of membranes—amniotic fluid**							
60 ng/mL	14/19 74% (95% CI 51–88)	147/151 97% (95% CI 93–99)	14/18 78% (95% CI 55–91)	147/152 97% (95% CI 93–99)	27.9 (95% CI 10.2–75.9)	0.27 (95% CI 0.13–0.57)	103 (95% CI 33–339)
**Preterm labor with intact membranes—amniotic fluid**							
120 ng/mL	7/12 58% (95% CI 32–81)	62/67 93% (95% CI 84–97)	7/12 58% (95% CI 32–81)	62/67 89% (95% CI 81–93)	7.8 (95% CI 3.0–20.6)	0.45 (95% CI 0.25–0.88)	17 (95% CI 3–64)
**Preterm prelabor rupture of membranes—cervical fluid**							
300 ng/mL	11/19 58% (95% CI 36–77)	145/151 96% (95% CI 92–98)	11/17 65% (95% CI 41–83)	145/153 95% (95% CI 90–97)	14.6 (95% CI 6.1–34.9)	0.44 (95% CI 0.26–74)	33 (95% CI 10–97)

CI: confidence interval.

#### PTL pregnancies

Differences in the concentrations of FcgammaBP were identified among the subgroups of women with intra-amniotic infection, sterile intra-amniotic inflammation, and negative amniotic fluid (infection: median 139.7 ng/mL, IQR 74.2–205.3; sterile: median 54.2 ng/mL, IQR: 44.8–127.0; negative: median 19.7 ng/mL, IQR: 15.9–23.6; Fig. 1b) in the crude analysis and after the adjustment for gestational age at sampling (both *p-*values < 0.0001). Women with intra-amniotic infection had higher amniotic fluid FcgammaBP concentrations than did women with sterile intra-amniotic inflammation and with negative amniotic fluid (Table 6). Women with sterile intra-amniotic inflammation had higher amniotic fluid FcgammaBP concentrations than those with negative amniotic fluid (Table 6).

**Table 6:** IgGFc-binding protein in amniotic fluid from preterm labor with intact membranes: the comparisons among the subgroups of the women with intra-amniotic infection, sterile intra-amniotic inflammation, and negative amniotic fluid.

	Intra-amniotic infection	Sterile intra-amniotic inflammation	Negative
Intra-amniotic infection	x	*p* = 0.04 **adj. p = 0.02**	*p* < 0.0001 **adj. p < 0.0001**
Sterile intra-amniotic inflammation	*p* = 0.04 **adj. p = 0.02**	x	*p* < 0.0001 **adj. p < 0.0001**
Negative	*p* < 0.0001 **adj. p < 0.0001**	*p* < 0.0001 **adj. p < 0.0001**	x

*p*-value: a comparison between two subgroups (a nonparametric Mann–Whitney *U* test);

Adj. *p*-value: a comparison between two subgroups after the adjustment for gestational age at sampling (a Spearman partial correlation).

Statistically significant results are marked in bold.

**Figure 3:**
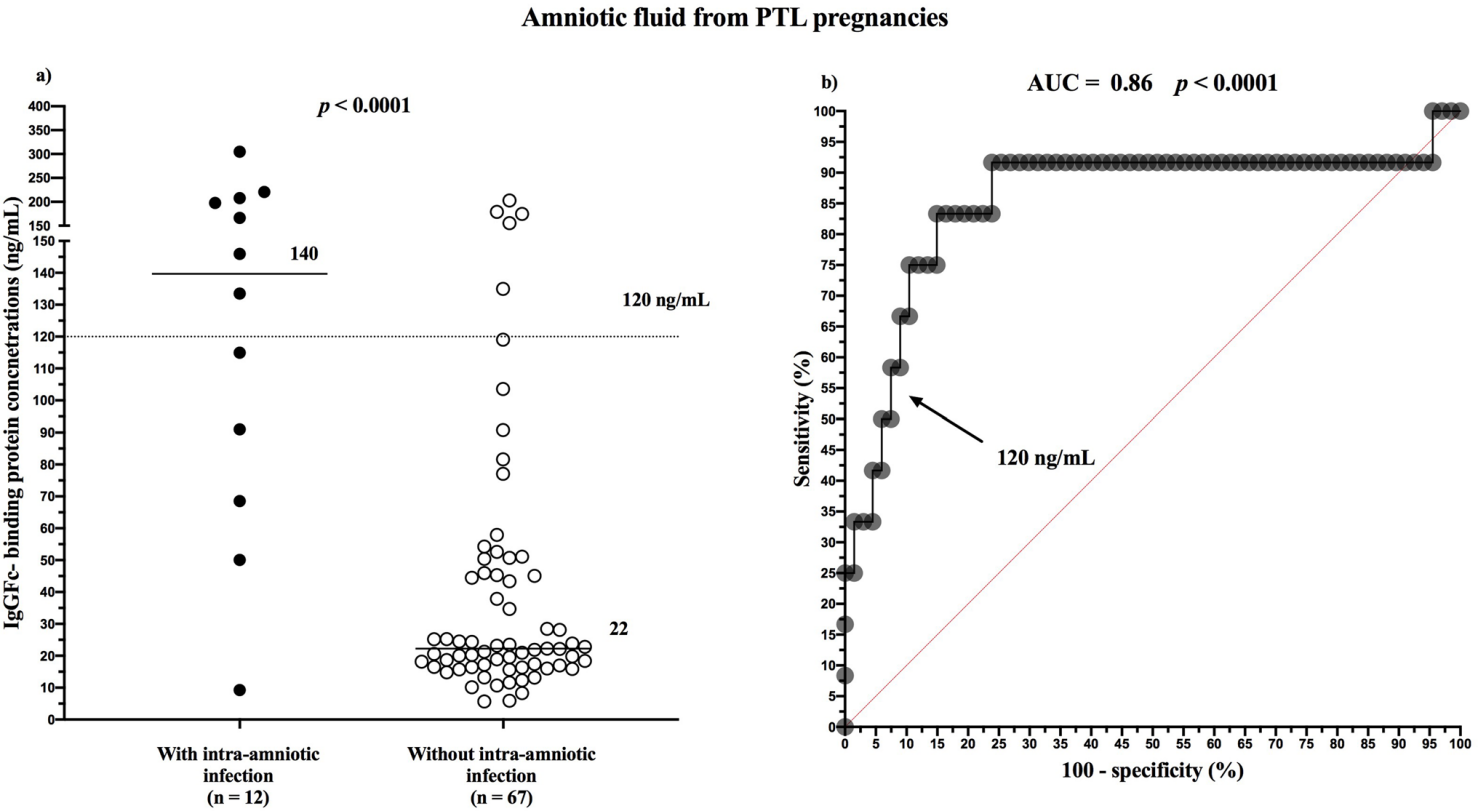
Amniotic fluid IgGFc-binding protein concentrations based on the presence of intra-amniotic infection in women with PTL (**a**) and receiver operating characteristic curves for amniotic fluid IgGFc-binding protein in women with PTL with intra-amniotic infection (**b**). PTL, preterm labor with intact membranes.

Women with intra-amniotic infection had higher concentrations of amniotic fluid FcgammaBP than those without intra-amniotic infection (with infection: median 139.7 ng/mL, IQR 74.2–205.3 vs. without infection: median 22.2 ng/mL, IQR 16.5–46.0; Fig. 3a). The amniotic fluid FcgammaBP cutoff value of 120 ng/mL was found to be optimal in the prediction of intra-amniotic infection (AUC = 0.86; *p* < 0.0001; Fig. 3b). The diagnostic indices of these cutoff values are in Table 5.

### Concentration of FcgammaBP in cervical fluid based on the phenotype of intra-amniotic inflammation

#### PPROM pregnancies

**Figure 4:**
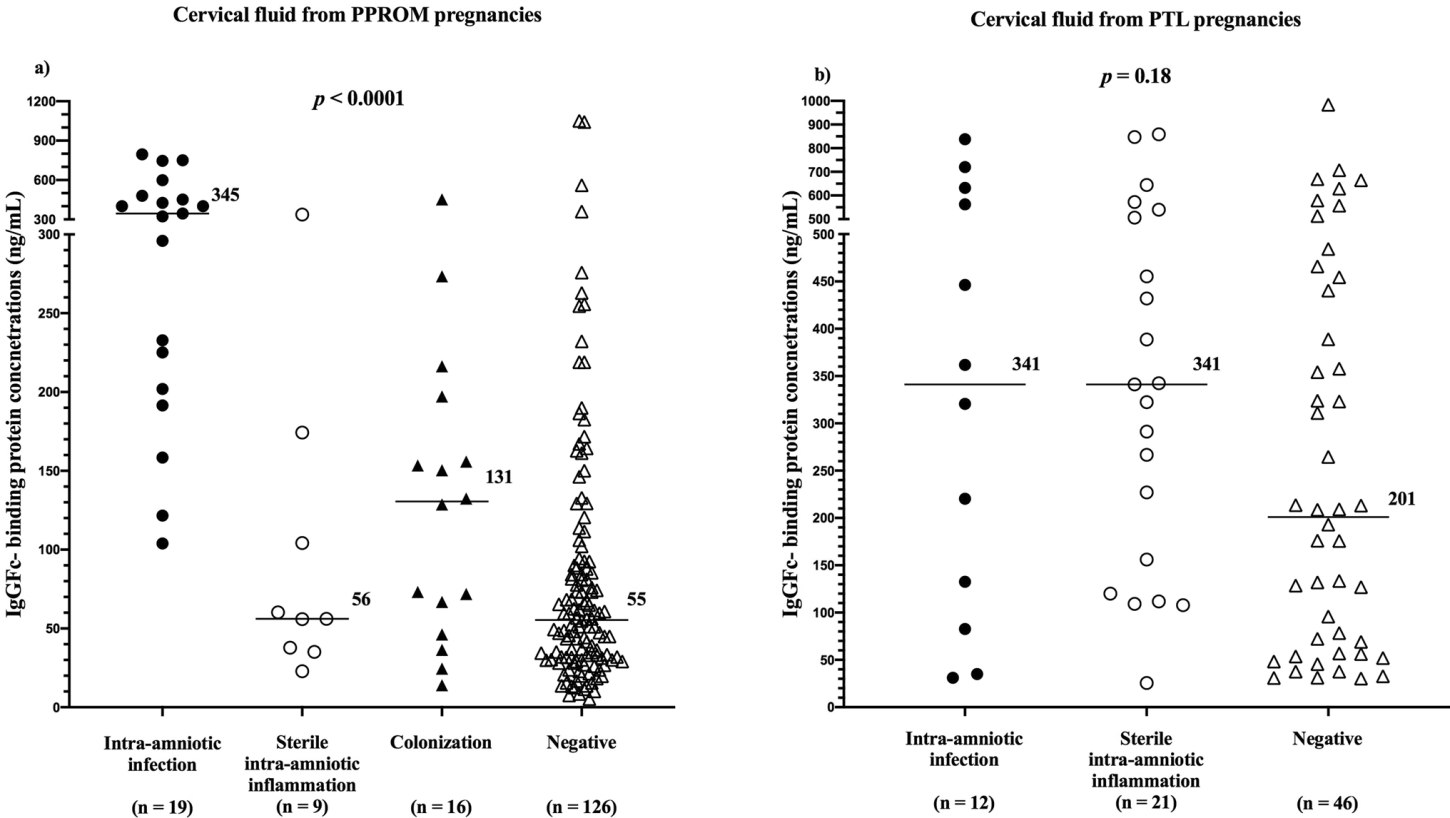
Cervical fluid IgGFc-binding protein concentrations in the subgroups of the women with PPROM (**a**) and with PTL (**b**). PPROM, preterm prelabor rupture of membranes; PTL, preterm labor with intact membranes.

A positive correlation was found between the concentrations of FcgammaBP in amniotic and cervical fluids (ρ = 0.34*; p* < 0.0001). The differences in cervical fluid FcgammaBP concentrations were revealed among the subgroups (infection: median 345.0 ng/mL, IQR 201.9–480.0; sterile: median 56.1 ng/mL, IQR 36.5–139.3; colonization: median 130.6 ng/mL, IQR 51.4–186.9; and negative: median 55.4 ng/mL, IQR 31.1–92.6; Fig. 4a) in the crude analysis as well as after the adjustment for gestational age at sampling (both *p* < 0.0001). Women with intra-amniotic infection had higher cervical fluid FcgammaBP concentrations than women with sterile intra-amniotic inflammation, colonization, and negative amniotic fluid (Table 7).

**Table 7:** IgGFc-binding protein in cervical fluid from preterm prelabor rupture of membranes: the comparisons among the subgroups of the women with intra-amniotic infection, sterile intra-amniotic inflammation, colonization, and negative amniotic fluid.

	Intra-amniotic infection	Sterile intra-amniotic inflammation	Colonization	Negative
Intra-amniotic infection	x	*p* = 0.0001 **adj. p = 0.001**	*p* < 0.0001 **adj. p < 0.0001**	*p* < 0.0001 **adj. p < 0.0001**
Sterile intra-amniotic inflammation	*p* = 0.0001 **adj. p = 0.001**	x	*p* = 0.28 adj. *p* = 0.76	*p* = 0.57 adj. *p* = 0.93
Colonization	*p* < 0.0001 **adj. p = 0.0001**	*p* = 0.28 adj. *p* = 0.76	x	*p* = 0.02 adj. *p* = 0.25
Negative	*p* < 0.0001 **adj. p < 0.0001**	*p* = 0.57 adj. *p* = 0.93	*p* = 0.02 adj. *p* = 0.25	x

*p*-value: a comparison between two subgroups (a nonparametric Mann–Whitney *U* test); adj. *p*-value: a comparison between two subgroups after the adjustment for gestational age at sampling (a Spearman partial correlation).

Statistically significant results are marked in bold.

**Figure 5:**
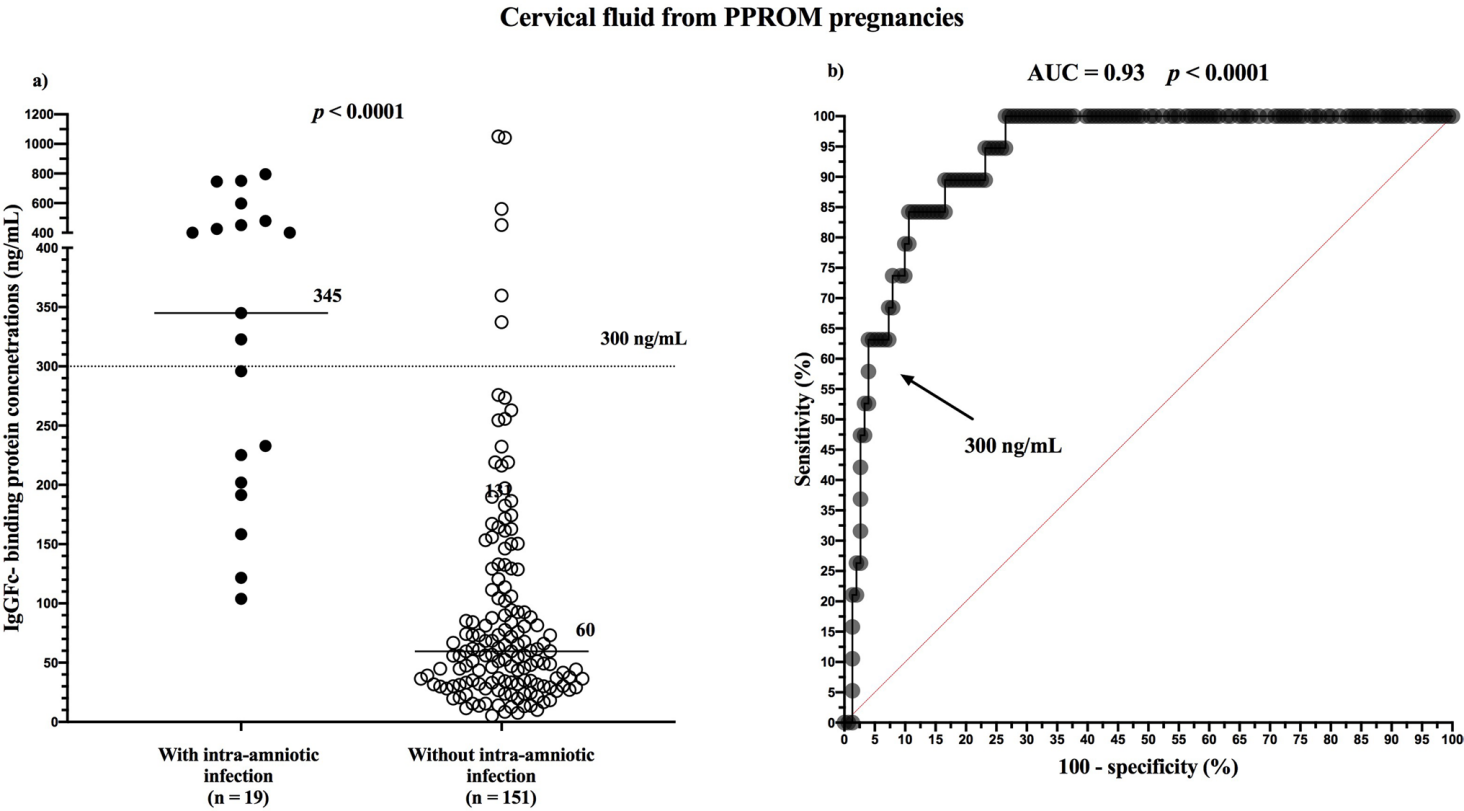
Cervical fluid IgGFc-binding protein concentrations based on the presence of intra-amniotic infection in women with PPROM (**a**) and receiver operating characteristic curves for cervical fluid IgGFc-binding protein in women with PPROM with intra-amniotic infection (**b**). PPROM, preterm prelabor rupture of membranes.

Women with intra-amniotic infection had higher cervical fluid FcgammaBP than those without intra-amniotic infection (with infection: median 345.0 ng/mL, IQR 201.9–480.0 vs. without infection: median 59.6 ng/mL, IQR 31.9–111.5; Fig. 5a). The cervical fluid FcgammaBP cutoff value of 300 ng/mL was found to be optimal in the prediction of intra-amniotic infection (AUC = 0.93; *p* < 0.0001; Fig. 5b). The diagnostic indices of these cutoff values are in Table 5.

#### PTL pregnancies

A weak positive correlation was observed between the concentrations of FcgammaBP in amniotic and cervical fluids (ρ = 0.25*; p* = 0.02). No difference in cervical fluid FcgammaBP concentrations was found among the subgroups (infection: median 341.1 ng/mL, IQR 95.2–614.8; sterile: median 341.2 ng/mL, IQR 138.1–523.4; and negative: median 200.9 ng/mL, IQR 56,7–443.8; *p* = 0.18; Fig. 4b). There was no difference in cervical fluid FcgammaBP concentrations between women with and without intra-amniotic infection (with infection: median 341.1 ng/mL, IQR 95.2–614.8 vs. without infection: median 227.0 ng/mL, IQR 95.7–455.4; *p* = 0.45).

## Discussion

### Principal findings of the study

(1) FcgammaBP was identified as a constituent of amniotic and cervical fluids from pregnancies complicated by PPROM and PTL; (2) the concentration of FcgammaBP in amniotic fluid was elevated in the presence of both phenotypes of intra-amniotic inflammation, being more pronounced in the presence of intra-amniotic infection in women with PTL; (3) the concentration of FcgammaBP in cervical fluid was elevated in the presence of intra-amniotic infection only in women with PPROM; (4) the FcgammaBP in amniotic fluid might be a marker of intra-amniotic infection in women with PPROM and PTL; and (5) the FcgammaBP in cervical fluid might be a non-invasive marker of intra-amniotic infection in women with PPROM.

### Meaning of the study

FcgammaBP was discovered more than 30 years ago as a specific site for the fragment of crystallizable (Fc) region of the immunoglobulin (Ig) G antibody in the small intestinal and colonic epithelia^65^. This specific site differed from previously recognized receptors in the Fc region of IgG^65^. The specific site for the Fc region of IgG was later termed FcgammaBP and identified as a protein primarily localized in the mucosal granules of the small intestinal and colonic epithelia that are secreted into the intestinal lumen^66^. Based on the current knowledge, FcgammaBP is considered to be a protein that provides immunologic protection of the intestinal tissue and facilitates the interaction between the intestinal mucus and potentially harmful stimuli (microorganisms, alarmins) with the ultimate goal of protecting the mucosal surface^62,65,66^. However, its exact biological function has yet to be fully elucidated.

The production of FcgammaBP has been described to occur in the intestinal epithelial cells, placenta, and thyroid tissue^62,63^. However, its expression has not been observed in the brain, heart, kidney, liver, lung, and skeletal muscles^62^. Interestingly, the ability to produce FcgammaBP was confirmed only in humans and monkeys, but not in mice, rats, rabbits, dogs, bovines, and porcines^62^.

FcgammaBP has been found in low concentrations in human serum from healthy individuals^64^. However, its serum concentrations were elevated in the presence of autoimmune diseases such as Crohn’s disease, ulcerative colitis, rheumatoid arthritis, systemic lupus erythematosus, and progressive systemic sclerosis^64^. The presence of FcgammaBP has been further proven in amniotic fluid, urine, saliva, and cerebrospinal fluid^40,42^. Liu et al. found FcgammaBP to be a constituent of amniotic fluid in the second trimester of uncomplicated pregnancies^42^. In addition, FcgammaBP was shown to be among the most abundant (35/1624) proteins found in amniotic fluid^42^. Our group described the amniotic fluid presence of FcgammaBP in pregnancies complicated by PPROM and PTL^36,37^. The observation from this study, where we found FcgammaBP as a constituent of amniotic fluid from pregnancies with PPROM and PTL, is in line with the abovementioned findings.

Previously, concentrations of FcgammaBP in amniotic fluid have been shown to be higher in women with PPROM with microbial invasion of the amniotic cavity and acute histological chorioamnionitis than in those without these complications^36^. Interestingly, no differences in the amniotic fluid concentration of FcgammaBP between the presence and absence of the abovementioned complications were identified in women with PTL, where amniotic fluid was obtained from the forewaters at the end of the first stage of labor^37^.

In this study, we found elevated amniotic fluid concentrations of FcgammaBP in the presence of both phenotypes of intra-amniotic inflammation. Interestingly, in women with PTL, the concentrations of FcgammaBP in amniotic fluid were higher in the presence of intra-amniotic infection than in the presence of sterile intra-amniotic inflammation. Collectively, the results from this study clearly show that both infectious and non-infectious stimuli might trigger the production of FcgammaBP.

In this study, the concentrations of FcgammaBP were measured in paired amniotic and cervical fluid samples obtained from women with both phenotypes of spontaneous preterm delivery. Interestingly, the FcgammaBP concentrations were higher in the cervical fluid samples than in the amniotic fluid samples, despite the fact that cervical fluid samples obtained with a swab were diluted in 1.5 mL of the buffer. These observations suggest that epithelial cells and/or immune cells in the endocervical canal are able to produce FcgammaBP. This finding supports the key role of the cervix during pregnancy, which is its immunologic protection against the ascension of microorganisms from the vagina and the cervix toward the upper genital tract^67–70^.

It is obvious that the protein composition of a cervical fluid sample from pregnancies with PPROM may be substantially affected by amniotic fluid that has leaked from the amniotic cavity. Therefore, the cervical fluid samples of PPROM pregnancies may reflect such a situation in both the intra-amniotic and cervical compartments. This is a possible explanation as to why women with intra-amniotic infection had higher cervical fluid FcgammaBP concentrations than those without intra-amniotic infection in PPROM but not in PTL pregnancies. However, a weak positive correlation between amniotic and cervical fluid FcgammaBP protein concentrations was also found in PTL.

This study suggests that FcgammaBP might be a new biomarker for intra-amniotic infection in both phenotypes of spontaneous preterm delivery. This finding is clinically very relevant since confirmation of intra-amniotic infection represents a challenge for clinicians. The necessity to rule in or rule out the presence of microorganisms in amniotic fluid makes the diagnosis of intra-amniotic infection time-consuming and more expensive when the techniques used to identify either non-culturable or difficult-to-culture microorganisms are employed. Therefore, from a clinical point of view, there is an urgent need to discover a single marker of intra-amniotic infection that has reliable sensitivity and specificity. In this study, FcgammaBP in amniotic fluid was identified as a potential marker of intra-amniotic infection in pregnancies with PPROM and PTL. Previously, Chaemsaithong et al. has described diagnostic indices of rapid matrix metalloproteinase (MMP)-8 and interleukin (IL)-6 point-of-care test (two cut-off values: 745 pg/mL and 1000 pg/mL) to identify intra-amniotic infection in pregnancies with PTL^21^. Comparing diagnostic indices among amniotic fluid FcgammaBP, MMP-8, and IL-6 to identify intra-amniotic infection in pregnancies with PTL, amniotic fluid FcgammaBP has the highest likelihood ratio [FcgammaBP (cut-off value of 120 ng/mL) 7.8, MMP-8 (cut-off value of 10 ng/mL) 3.3, IL-6 (cut-off value of 745 pg/mL) 2.6, and IL-6 (cut-off value of 1000 pg/mL) 3.0]^21^.

In addition, in women with PPROM, FcgammaBP in cervical fluid was also revealed as a potential marker of intra-amniotic infection. Particularly, cervical fluid FcgammaBP can be a clinically relevant marker given the non-invasive nature of cervical fluid sampling. Moreover, its diagnostic indices are better than previously published diagnostic indices of IL-6 in cervical fluid (AUC = 0.78, the positive likelihood ratio of 4.8) in PPROM pregnancies^71^. In addition, cervical fluid sampling can be safely repeated during the latency period of pregnancy complicated by PPROM. Therefore, FcgammaBP assessment in cervical fluid might be used to monitor the development of secondary intra-amniotic infection during the latency interval in women with PPROM.

### Strengths and limitations of the study

The strength of this study is the relatively large cohort of paired samples of amniotic and cervical fluid. Second, the fluid samples were collected from well-defined phenotypes of spontaneous preterm delivery (PPROM and PTL). Finally, the thorough assessment of microbial invasion of the amniotic cavity, by a combination of culture and non-culture methods, provided an opportunity to precisely distinguish the subsets of women with intra-amniotic infection and sterile intra-amniotic inflammation.

This study also has some limitations that are worth mentioning. For example, there was a small number of women with intra-amniotic infection (n = 19 and n = 12). To confirm whether the concentration of FcgammaBP in amniotic fluid and cervical fluid is a reliable marker of intra-amniotic infection, the results need to be replicated in independent cohorts. Next, despite the FcgammaBP expression in the placenta that was described^62^, the questions of which part of the placenta is a source of FcgammaBP and whether fetal membranes produce FcgammaBP still remain unanswered. A body of evidence has shown that intestinal epithelial cells produce FcgammaBP (97, 102, 103), but no data are available on whether amniotic epithelial cells can produce FcgammaBP. Given the importance of the amniotic epithelium as a barrier against the ascension of microorganisms into the amniotic cavity^72–74^, some similarities between intestinal and amniotic epithelial cells might be identifiable such as, (1) to serve as mechanical barriers^72–76^; (2) to have spatially expressed toll-like receptors^77,78^; and (3) to indicate that the expression of toll-like receptors changes when inflammation is present^77–79^. Therefore, we hypothesize that the amniotic epithelium might be involved in FcgammaBP production.

## Conclusion

The concentrations of FcgammaBP observed in amniotic and cervical fluid were elevated in women with intra-amniotic infection. Thus, after replication in an independent cohort, FcgammaBP in amniotic fluid might be a potential marker of intra-amniotic infection in pregnancies with PPROM and PTL. Moreover, FcgammaBP in cervical fluid could be a marker of intra-amniotic infection in pregnancies with PPROM.

## Methods

This retrospective cohort study included pregnant women who were admitted to the Department of Obstetrics and Gynecology at the University Hospital Hradec Kralove in the Czech Republic between March 2017 and May 2020. The inclusion criteria were the following: (1) singleton pregnancy, (2) maternal age ≥ 18 years, (3) gestational age between 22 + 0 and 36 + 6 weeks, (4) PPROM or PTL, and (5) the performance of transabdominal amniocentesis at the time of admission to determine intra-amniotic inflammation. In contrast, the exclusion criteria were the following: (1) pregnancy-related and other medical complications such as fetal growth restriction, gestational or pre-gestational diabetes, gestational or chronic hypertension, and preeclampsia; (2) structural or chromosomal fetal abnormalities; (3) signs of fetal hypoxia; and (4) significant vaginal bleeding.

The gestational age was determined via the use of first-trimester fetal biometry. PPROM was diagnosed by examining the women, using a sterile speculum, for pooling of amniotic fluid in the posterior fornix of the vagina. In the case of clinical uncertainty in diagnosing PPROM, amniotic fluid leakage was confirmed by the presence of insulin-like growth factor-binding proteins (Actim PROM test; Medix Biochemica, Kauniainen, Finland) in the vaginal fluid.

PTL was diagnosed as the presence of regular uterine contractions (at least two contractions every 10 min), along with cervical length, measured using transvaginal ultrasound, shorter than 15 mm or within the 15–30 mm range with a positive PartoSure test (Parsagen Diagnostics Inc., Boston, MA)^80^.

Women with PPROM were treated with antibiotics. Those with intra-amniotic inflammation received first-line treatment with intravenous clarithromycin for seven days. Unless delivery occurred earlier, the antibiotic treatment was eventually modified under the condition of microbial invasion of the amniotic cavity; the women without intra-amniotic inflammation received benzylpenicillin (clindamycin was used in women allergic to penicillin). Women with PPROM below the gestational age of 35 + 0 weeks received corticosteroids (betamethasone) to accelerate fetal lung maturation and reduce neonatal mortality and morbidity. Women with PPROM were managed expectantly, except those with intra-amniotic infection beyond the gestational age 28 + 0 weeks wherein labor was induced or an elective cesarean section was performed within 72 h of admission.

Women with PTL received a course of corticosteroids (betamethasone) and tocolytic therapy with either intravenous atosiban (for gestational age ≤ 28 weeks) or with nifedipine, which was administered orally, for 48 h. Patients with proven intra-amniotic inflammation received treatment with intravenous clarithromycin for seven days, unless delivery occurred earlier. Antibiotic treatment was eventually modified under the condition of microbial invasion of the amniotic cavity. Women with PTL that were positive for group B Streptococcus (GBS), as determined from the vaginal-rectal swab, or with an unknown GBS status received intravenous benzylpenicillin (clindamycin, in case of penicillin allergy) during an active labor.

All participants in this study provided informed written consent prior to the collection of amniotic and cervical fluid samples. Sample collection for this research was approved by the Institutional Review Board of the University Hospital Hradec Kralove (July 2014; No. 201408 S07P). All experiments were performed in accordance with relevant guidelines and regulations. All participants were Caucasian.

### Cervical and amniotic fluid sampling

Paired cervical fluid and amniotic fluid samples were collected at the time of admission from all women included in this study, prior to the administration of antibiotics, tocolytics, and/or corticosteroids. Each cervical fluid sample was obtained by placing a Dacron polyester swab in the cervical canal for 20 s to achieve saturation. Once collected, the polyester swab was inserted into a polypropylene tube containing 1.5 mL of phosphate-buffered saline; the tube was then shaken for 20 min. Upon removal of the polyester swab, the tube was centrifuged at 300×*g* for 15 min at room temperature. The supernatant was divided into aliquots and stored at − 80** °C** until further analysis.

Ultrasonography-guided transabdominal amniocentesis was performed after cervical fluid sampling. Approximately 2–3 mL of amniotic fluid was aspirated, and the amniotic fluid was immediately divided among polypropylene tubes. The samples of amniotic fluid were used for (i) the assessment of amniotic fluid interleukin (IL)-6; (ii) polymerase chain reaction (PCR) analysis of *Ureaplasma* species, *Mycoplasma hominis*, and *Chlamydia trachomatis*; (iii) sequencing of the 16S rRNA gene; and (iv) aerobic and anaerobic cultivation.

### Amniotic fluid IL-6 concentrations

IL-6 concentrations were assessed using the immuno-analyzer Cobas e602, a part of the Cobas 8000 platform (Roche Diagnostics, Basel, Switzerland). The measurement range was 1.5–50,000 pg/mL. The coefficient of inter- and intra-assay precision was < 10%^22^.

### Detection of *Ureaplasma* species, *M. hominis*, and *C. trachomatis*

DNA was isolated from amniotic fluid using the QIAamp DNA Mini Kit (Qiagen, Hilden, Germany), according to the manufacturer’s instructions. Real-time PCR was conducted on a Rotor-Gene 6000 instrument (Qiagen) using the commercial AmpliSens *C. trachomatis*/*Ureaplasma*/*M. hominis*-FRT kit (Federal State Institution of Science, Central Research Institute of Epidemiology, Moscow, Russia) to detect the DNA from *Ureaplasma* species, *M. hominis*, and *C. trachomatis* in the same PCR tube (multiplex format). We included a PCR run for beta-actin, a housekeeping gene that served as the control, to examine the presence of polymerase chain reaction inhibitors.

### Detection of other bacteria in amniotic fluid

Bacterial DNA was identified by PCR targeting the 16S rRNA gene with the following primers: 5′-CCAGACTCCTACGGGAGGCAG-3′ (V3 region) and 5′-ACATTTCACAACAC-GAGCTGACGA-3′ (V6 region)^81,82^. Each reaction contained 3 μL of target DNA, 500 nM forward and reverse primers, and Q5 High-Fidelity DNA polymerase (NEB, Ipswich, MA, USA) in a total volume of 25 μL. Amplification was carried out on a 2720 Thermal Cycler (Applied Biosystems, Foster City, CA, USA). The products were visualized on an agarose gel. Positive reactions yielded 950 bp products that were subsequently analyzed by sequencing. The 16S rDNA PCR products were purified and subjected to sequencing with the above-mentioned primers and the BigDye Terminator kit v.3.1 (Thermo Fisher Scientific, Waltham, MA, USA). The bacteria were then typed via searches for the obtained sequences using BLAST and SepsiTest BLAST.

### Aerobic and anaerobic cultures of amniotic fluid

The amniotic fluid samples were cultured on Columbia agar with sheep’s blood, *Gardnerella vaginalis* selective medium, MacConkey agar, a *Neisseria*-selective medium (modified Thayer–Martin medium), Sabouraud agar, or Schaedler anaerobe agar. The plates were cultured for 6 days and checked daily. The species were identified by matrix-assisted laser desorption/ionization time-of-flight mass spectrometry using MALDI Biotyper software (Bruker Daltonics, Bremen, Germany).

### Clinical definitions

*Microbial invasion of the amniotic cavity* was determined based on a positive PCR analysis of *Ureaplasma* species*, M. hominis, C. trachomatis* or a combination of these species or positivity for the 16S rRNA gene, aerobic/anaerobic cultivation of the amniotic fluid, or a combination of these parameters. *Intra-amniotic inflammation* was defined as amniotic fluid IL-6 concentrations ≥ 3000 pg/mL^22^. *Intra-amniotic infection* was defined by both microbial invasion of the amniotic cavity and intra-amniotic inflammation. *Sterile intra-amniotic inflammation* was defined as the presence of intra-amniotic inflammation without the concomitant microbial invasion of the amniotic cavity. *Colonization* was defined as the microbial invasion of the amniotic cavity without intra-amniotic inflammation. *Negative amniotic fluid* was defined as the absence of microbial invasion of the amniotic cavity and intra-amniotic inflammation.

### Quantification of FcgammaBP in amniotic and cervical fluids

The concentrations of FcgammaBP were assessed in the amniotic fluid and cervical fluid samples using an enzyme-linked immunosorbent assay (ELISA), the Human FCGBP ELISA Kit (LifeSpan BioSciences, Inc., Seattle, WA, USA), according to the manufacturer’s instructions. The amniotic fluid and cervical fluid samples were diluted tenfold and 50-fold, respectively. The sensitivity of the kit was 0.117 ng/mL. The absorbance values were read at 450 nm on a Multiskan RC ELISA reader (Thermo Fisher Scientific).

### Statistical analyses

The women’s demographic and clinical characteristics were compared using the nonparametric Mann–Whitney *U* test for continuous variables and are presented as median values (interquartile range [IQR]). Categorical variables were compared using Fisher’s exact test and are presented as a number (%). The normality of the data was tested using the Anderson–Darling test. Because the FcgammaBP concentrations in the amniotic fluid were not normally distributed, the nonparametric Kruskal–Wallis and Mann–Whitney *U* tests were performed for statistical analyses, and the results are presented as a median value (IQR). Spearman’s partial correlation analysis was performed to adjust the results for gestational age at sampling. Spearman’s correlation was used to assess the relationship between the concentrations of amniotic fluid FcgammaBP and cervical fluid FcgammaBP and gestational age at sampling. Receiver operating characteristic (ROC) curves were constructed to assess the predictive value of amniotic fluid and cervical fluid FcgammaBP for the presence of intra-amniotic infection. Cutoff values were determined based on the highest positive likelihood ratio. Differences were considered significant at *p* < 0.05. All *p* values were obtained using two-tailed tests, and all statistical analyses were performed using GraphPad Prism, version 8.1.1. for Mac OS X (GraphPad Software, San Diego, CA, USA) or the Statistical Package for Social Sciences (SPSS), version 19.0 for Mac OS X (SPSS Inc., Chicago, IL, USA).
